# Mechanism of SCN^−^ ions affecting the adsorption of Au(CN)_2_^-^ ions on anion exchange resin

**DOI:** 10.3389/fchem.2026.1908266

**Published:** 2026-07-30

**Authors:** Yinyu Ma, Haiyun Xie, Wang Jiang, Liuyang Dong, Zixin Song, Xiaotong Huo, Dianwen Liu

**Affiliations:** 1 State Key Laboratory of Complex Nonferrous Metal Resources Clean Utilization, Yunnan Key Laboratory of Green Separation and Enrichment of Strategic Mineral Resources, Faculty of Land Resources Engineering, Kunming University of Science and Technology, Kunming, China; 2 China Southwest United Graduate School, Kunming, China; 3 Chifeng Chaihu Lanzi Gold Mining Co., LTD, Chifeng, Inner Mongolia, China

**Keywords:** adsorption mechanism, gold cyanide complex ion, resin, resin-in-pulp process, thiocyanate ion

## Abstract

The cyanide leaching process for gold ore is a mainstream gold extraction method due to its high gold leaching efficiency and strong ore adaptability. The resin-in-pulp process is an important cyanidation-based gold recovery technique, which employs weakly basic anion exchange resins with high selectivity and large adsorption capacity as adsorbents, offering the significant advantage of high adsorption efficiency. However, in practical production, repeated resin recycling accumulates SCN^−^ in pulp from residual thiocyanate desorption released during each reuse, significantly reducing Au(CN)_2_^-^ adsorption efficiency. To address this issue, this study focuses on the macroporous weakly basic anion exchange resin D301 used in a gold mine in Chifeng, Inner Mongolia, China. Through single-factor tests and comparative experiments, the influence of SCN^−^ ions on the adsorption of Au(CN)_2_^-^ by the resin was investigated. Experimental results indicate that under conditions where SCN^−^ ions are present, increasing the experimental temperature or initial solution pH reduces the adsorption efficiency of Au(CN)_2_^-^, whereas increasing the resin dosage effectively enhances adsorption. Microscopic characterization further elucidates the mechanism by which SCN^−^ affects the adsorption process. SEM-EDS analysis reveals that SCN^−^ diminishes the resin’s adsorption capacity for Au(CN)_2_^-^. FTIR spectra show the simultaneous presence of characteristic peaks for Au(CN)_2_^-^ (C≡N) and SCN^−^ (S–C≡N) on the resin surface, confirming their coexistence. XPS results further verify the existence of characteristic peaks for Au and S elements, indicating that while gold is adsorbed in the form of Au(CN)_2_^-^, the resin also adsorbs SCN^−^ from the solution. In summary, SCN^−^ and Au(CN)_2_^-^ exhibit a competitive adsorption mechanism on D301 resin, which is the primary cause of the decreased adsorption efficiency of Au(CN)_2_^-^. It is recommended that when the concentration of SCN^−^ in the pulp accumulates to a high level during the resin-in-pulp gold extraction process, it should be removed by oxidation, thereby enabling efficient adsorption of Au(CN)_2_^-^ by the resin. This study provides both theoretical and experimental foundations for addressing SCN^−^ interference and optimizing resin-based gold extraction processes.

## 1 Introduction

Gold is a critical strategic metal widely utilized in aviation, medical, electronic, and chemical industries. As high-grade and easily accessible gold ores become increasingly depleted, low-grade and complex ores have progressively become the primary sources for gold production (Atefeh et al., 2021; Han et al., 2024; Kenzhaliyev et al., 2023; Zhang et al., 2018). The cyanide leaching process remains the dominant method for gold extraction worldwide, owing to its technical maturity, high recovery efficiency, and strong adaptability to various ore types, accounting for over 90% of industrial gold production (Han et al., 2020; Surimbayev et al., 2024; Zhong et al., 2018).

Among the various cyanidation-based processes, the resin-in-pulp method is particularly advantageous for treating low-grade and complex ores, as it simplifies the operational workflow, eliminates the need for solid-liquid separation, and offers high adsorption efficiency (Aylmore and Muir, 2003; Kotze and Wyethe, 2000; Laverdiev et al., 2006). In this process, the performance of anion exchange resins directly determines the gold adsorption efficiency. D301, a macroporous weakly basic anion exchange resin, has been widely adopted by numerous gold production enterprises in China due to its high mechanical strength, favorable adsorption capacity, and ease of regeneration (Liu and Wang, 1991; Niu et al., 2024; Zhang et al., 1991).

Nevertheless, in actual production practice, high concentrations of SCN^−^ often accumulate in the leaching system, severely impairing the resin’s adsorption performance for Au(CN)_2_^-^. SCN^−^ is primarily derived from two sources: the oxidation of sulfide minerals (e.g., pyrite) present in the ore during cyanidation, and the use of thiocyanate salts as eluants for gold-loaded resins, which leads to continuous SCN^−^ build-up in the circulating solution (Atefeh et al., 2023; Lin et al., 2025; Rabieh et al., 2017; Yonghui et al., 2021). To evaluate the actual impact of SCN^−^, a compositional analysis of the circulating water from a gold plant in Chifeng, Inner Mongolia, China, was conducted in this study. The results revealed a strongly alkaline solution (pH = 12.16), with SCN^−^ as the predominant anion at a concentration of 4,175.74 mg/L, accompanied by 1845.00 mg/L of free cyanide (CN^−^). Under these conditions, the resin’s Au(CN)_2_^-^ adsorption efficiency was only 86.67%, resulting in significant gold losses and increased economic burden.

Current methods for SCN^−^ removal include the alkali-chlorine process, ozone oxidation, hydrogen peroxide oxidation, and biodegradation (Tae-Yoon et al., 2004; Ashby et al., 2004). Among these, the H_2_O_2_ oxidation method shows promising application prospects due to its thorough reaction, lack of secondary pollution, and operational simplicity (Oulego et al., 2014). However, most existing studies have focused on the treatment of SCN^−^-containing wastewaters, with limited attention given to its effects and mechanisms in resin adsorption systems-particularly regarding the adsorption behavior of Au(CN)_2_^-^ onto resins in the presence of SCN^−^ (Cakiroglu et al., 2025; Guo et al., 2025; Shi and Yu, 2025; Wang et al., 2025).

Therefore, this study employs D301 resin to investigate the influence of SCN^−^ on the resin adsorption of Au(CN)_2_^-^. Through single-factor and comparative experiments, the patterns of SCN^−^ effects are systematically examined, and the underlying mechanisms are analyzed using SEM-EDS, FTIR, and XPS techniques. The findings are expected to provide theoretical foundations and technical references for optimizing resin adsorption processes in SCN^−^-containing cyanide systems and for effectively controlling SCN^−^ in industrial production.

## 2 Materials and methods

### 2.1 Test materials and reagents

The macroporous weakly basic polystyrene anion exchange resin (D301) employed in this work was provided by the Chifeng Gold Mine. The gold standard solution and other reagents (NaOH, HCl, and H_2_SO_4_) were sourced from Shanghai Aladdin Bio-Chemical Technology Co., Ltd., all of analytical grade. Detailed characterization of the resin is given in Section 2.1.1.

#### 2.1.1 Analysis of the properties of D301 resin

##### 2.1.1.1 Physical properties and appearance

**FIGURE 1:**
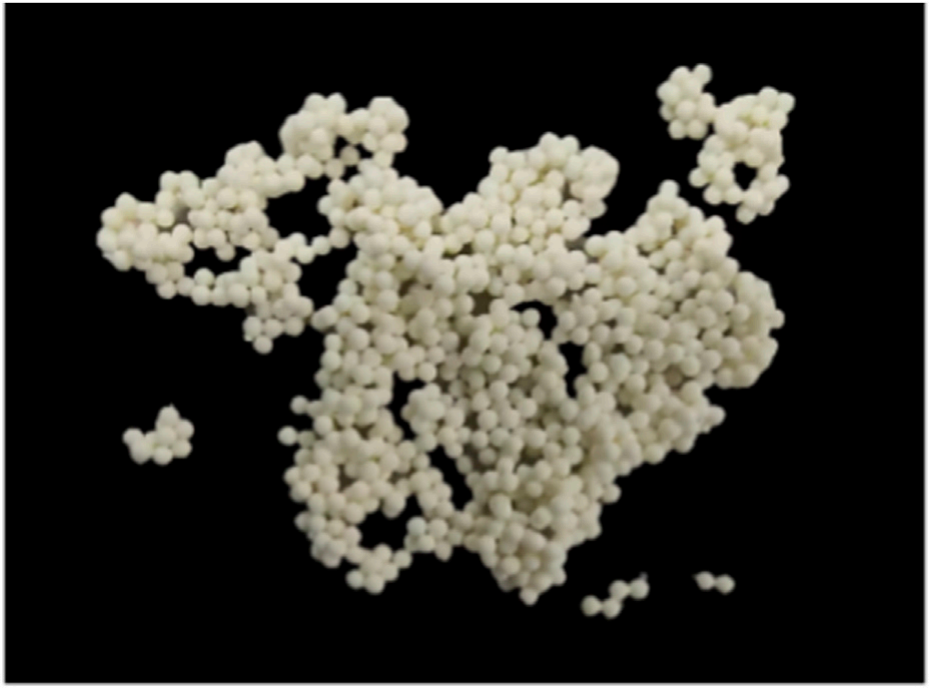
Photograph of D301 resin.

The key performance parameters of D301 resin are summarized in Table 1. The resin appears as milky white, opaque spherical particles free from visible impurities (Figure 1).

**TABLE 1:** Key performance parameters of D301 resin.

Category	D301 resin
Matrix structure	Polystyrene
Functional group	$-NR_{2}^{\prime },=N^{+}R_{2}^{\prime }$
Total exchange volume	≥4.2 mmol/g dry resin
	≥1.1 mmol/g wet resin
Strong-base exchange capacity	≥0.9 mmol/g wet resin
Moisture content	55%∼65%
Appearance	Milky white or light yellow opaque spherical particles
Particle size	1.0∼1.6 mm (≥95%)
Moisture-adjusted density	1.03∼1.07 g/mL
Wet apparent density	0.59∼0.69 g/mL
Ball rate after grinding	≥95%
Maximum service temperature	40 °C (salt form) 100 °C (free base form)
pH range of use	1–12
Factory type	Free base
Resin saturated gold capacity (CN^−^ 2000 mg/L, Au(CN)_2_^–^100 mg/L, pH 12, 20 °C, resin dosage 1 g/L)	32.20 mg/g

As shown in Table 1, D301 is a polystyrene resin with high exchange capacities, indicating strong Au(CN)_2_^-^ uptake potential. Its moisture content (55%–65%) reflects high porosity, which facilitates ion access and exchange kinetics, thereby enhancing adsorption rate and capacity. The resin also exhibits low density, good mechanical strength (grinding ball rate ≥95%), broad pH tolerance (1–12), and a high maximum service temperature (100 °C in free base form), making it well-suited for the resin-in-pulp process.

##### 2.1.1.2 Scanning electron microscopy (SEM) analysis

**FIGURE 2:**
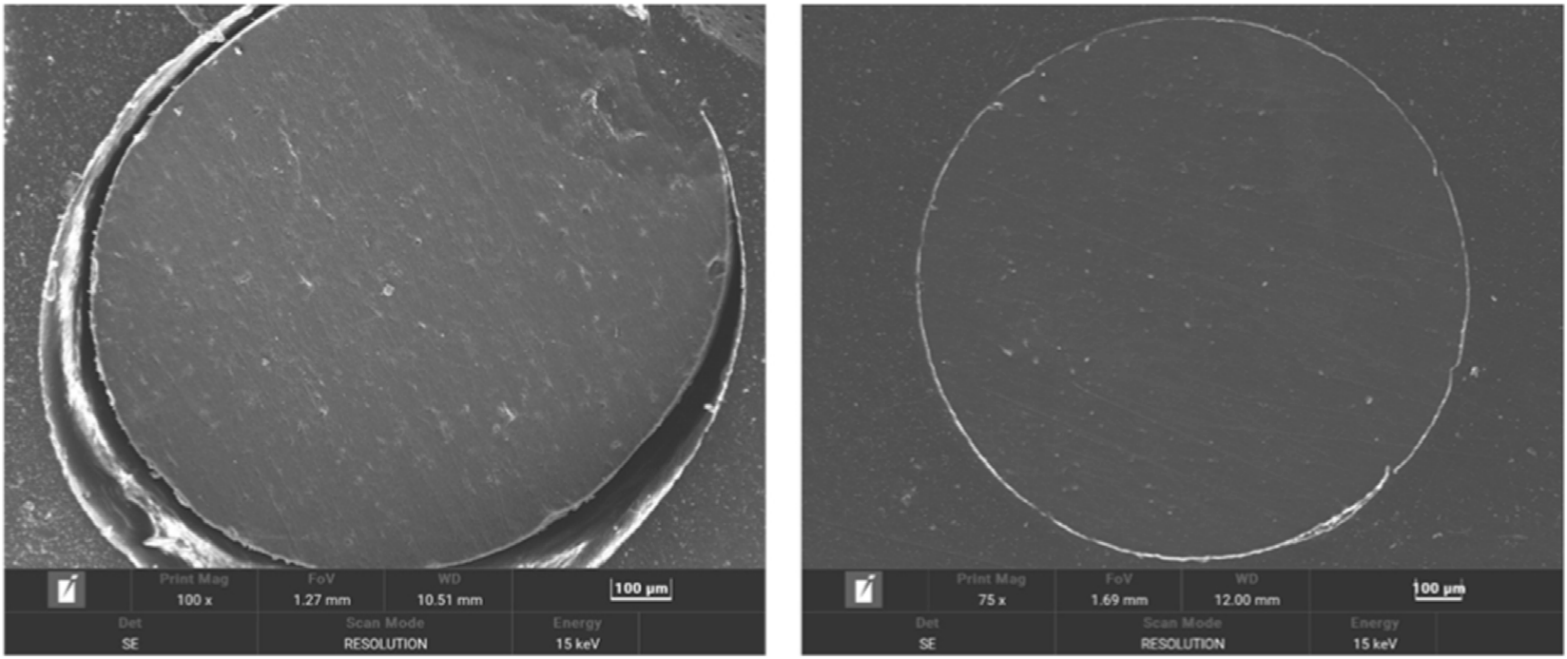
SEM analysis results of D301 resin.

**FIGURE 3:**
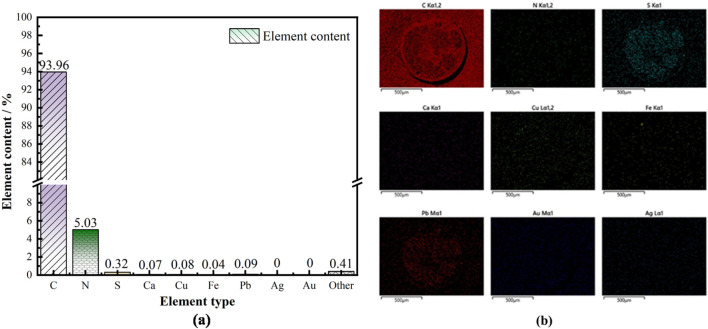
Elemental content and distribution of D301 resin. **(a)** D301 Resin Elements Content. **(b)** Distribution of Elements in the D301 Resin.

The surface morphology of the D301 resin microstructure was examined using a MIRA LMS scanning electron microscope, and semi-quantitative elemental analysis was performed with an Xplore 30 energy-dispersive spectrometer. The microstructure and elemental composition are shown in Figures 2, 3, respectively.

As shown in Figure 2, the resin particles exhibit intact spherical morphology with smooth and uniformly distributed cross-sectional edges, and no internal cracks are observed. Figure 3 reveals that the resin surface is predominantly composed of carbon (93.96%) and nitrogen (5.03%), with only trace amounts of Ca, Cu, and Fe, indicating that no significant metal ion contamination occurred during resin manufacturing. Notably, no Au or Ag was detected on the resin surface, confirming that the resin had not undergone any prior adsorption process.

##### 2.1.1.3 FTIR analysis

**FIGURE 4:**
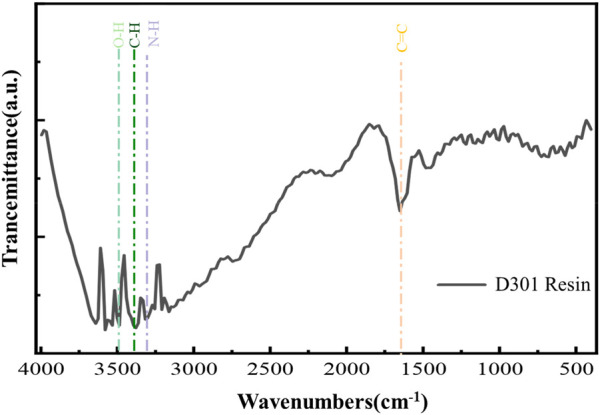
Analysis of FTIR test results of D301 resin.

The functional groups of the resin were further characterized by Fourier transform infrared spectroscopy (FTIR). The spectrum is shown in Figure 4.

The FTIR spectrum exhibits multiple absorption bands in the 3,100–3,700 cm^−1^ region. The band at approximately 3,450 cm^−1^ is characteristic of the N–H stretching vibration associated with the tertiary amine groups present in the resin matrix. A strong absorption at 1,650.77 cm^−1^ is attributed to the C=C stretching vibration of the aromatic ring in the polystyrene backbone. These features confirm the presence of abundant tertiary amine functional groups in the D301 resin.

##### 2.1.1.4 Determination of saturated gold loading

**FIGURE 5:**
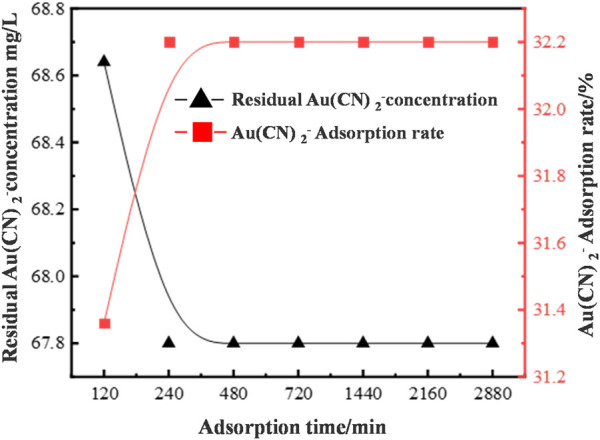
Determination results of saturated adsorption capacity of D301 resin for Au (CN)_2_^-^.

The saturated adsorption capacity of D301 resin for Au(CN)_2_^-^ was determined under simulated production conditions (CN^−^ 2000 mg/L, Au(CN)_2_^–^100 mg/L, pH 12, 20 °C, resin dosage 1 g/L). Figure 5 shows that adsorption reached equilibrium at 480 min with a loading of 32.20 mg/g (adsorption efficiency 32.20%), which serves as a reference for later solution preparation.

The saturated adsorption capacity of D301 resin for Au(CN)_2_^-^ obtained in this study (32.20 mg/g) is comparable to values reported for similar weak-base anion exchange resins in cyanide systems, which typically range from 16 to 50 mg/g depending on experimental conditions (An et al., 2016; Niu et al., 2024).

### 2.2 Preparation of standard adsorption solutions and analytical procedures

An artificially prepared standard adsorption solution was used, containing 2000 mg/L CN^−^ and 35 mg/L Au(CN)_2_^-^. For a 1 L preparation, deionized water was adjusted to alkaline pH with NaOH, then 3.77 g of NaCN was added and dissolved completely. A calculated volume of 1,000 μg/mL gold standard solution was spiked to achieve the desired Au(CN)_2_^-^ concentration, and the final pH was readjusted to 12.

The Au(CN)_2_^-^ concentration in the solution was determined using an Agilent 5110 ICP-OES at the characteristic gold emission wavelength of 329 nm, where the line intensity is proportional to the element concentration. The sample was nebulized and carried by argon into the plasma, where high temperature and a high-frequency electromagnetic field excite the characteristic atomic spectra, enabling quantitative analysis based on spectral intensity.

The adsorption rate (R) of D301 resin for Au(CN)_2_^-^ was calculated using the following Equation 1:

$$R=\left(C‐C^{\prime }\right)/C\times 100\%$$ (1)

where R is the adsorption efficiency (%), *C* is the initial concentration of Au(CN)_2_^-^ in the solution before adsorption (mg/L), and *C′* is the concentration of Au(CN)_2_^-^ remaining in the solution at adsorption time (mg/L). All concentrations were determined by ICP-OES as described above.

### 2.3 SCN^−^ concentrations determination

The determination of SCN^−^ concentration follows the standard HG/T 6072-2022. The instrument used was a TU-1900 UV-Vis spectrophotometer (Beijing Puxi General Instrument Co., Ltd.). Cations were eluted isocratically with oxalic acid as the mobile phase, while anions were eluted isocratically with sodium carbonate/sodium bicarbonate as the mobile phase. After eluting sample impurities, the eluate was diluted for SCN^−^ concentration measurement.

### 2.4 Scanning electron microscope (SEM) analysis

The surface morphology of D301 resin particles before adsorption was examined using a MIRA LMS scanning electron microscope (SEM, TESCAN, Czech Republic). Semi-quantitative elemental analysis of the resin surface was performed using an Xplore 30 energy-dispersive X-ray spectrometer (EDS, Oxford Instruments, United Kingdom).

For comparative analysis, three resin samples were prepared: (1) fresh D301 resin before adsorption; (2) D301 resin after adsorption in SCN^−^-free solution; and (3) D301 resin after adsorption in SCN^−^-containing solution. Each sample was ground into powder and sieved through a 400-mesh standard sieve. The sieved powders were then characterized using a Sigma 300 field-emission SEM (ZEISS, Germany) equipped with an Xplore 30 EDS (Oxford Instruments, United Kingdom) for surface morphology observation and semi-quantitative elemental analysis.

### 2.5 X-ray diffraction (XRD) analysis

The D301 resin before adsorption, the D301 resin after adsorption without SCN^−^ in the solution, and the D301 resin after adsorption in the presence of SCN^−^ in the solution were developed into powders, respectively. The 400-mesh standard sieve was used for screening, and several grams of the resin powder sample of-400 mesh were taken. The main chemical phases of the resin and other samples before and after adsorption were analyzed by Ultima IV X-Ray Diffraction (XRD) produced by Rigaku Company in Japan.

### 2.6 Fourier infrared spectroscopy analysis

The D301 resin before adsorption, the D301 resin after adsorption without SCN^−^ in the solution, and the D301 resin after adsorption in the presence of SCN^−^ in the solution were developed into powders, respectively. The 400-mesh standard sieve was used for screening. Several grams of resin powder samples with 400 mesh were taken and prepared by potassium bromide tabletting method. The Tensor 27 Fourier Transform Infrared Spectroscopy (FTIR) produced by the German Bruker company was used to test the functional groups in the sample in the wavelength range of 4,000–400 cm^−1^.

### 2.7 X-ray photoelectron spectroscopy analysis

The D301 resin before adsorption, the D301 resin after adsorption without SCN-in the solution, and the D301 resin after adsorption in the presence of SCN-in the solution were developed into powders, respectively. The 400-mesh standard sieve was used for screening, and several grams of the resin powder sample of-400 mesh were taken. The K-Alpha X-ray Photoelectron Spectrometer (XPS) produced by Thermo Fisher Scientific was used to analyze the valence state of specific elements on the surface of the sample and their possible material composition.

### 2.8 Oxidation test of SCN^−^ with H_2_O_2_

The oxidative removal of high-concentration SCN^−^ from the solution using hydrogen peroxide is an environmentally friendly process. The experiment was conducted at 30 °C. A certain volume of the solution to be oxidized and a certain volume of hydrogen peroxide solution (containing 30% H_2_O_2_) were measured separately, and then the hydrogen peroxide was slowly added to the solution to be oxidized. After a certain reaction time, the residual SCN^−^ concentration in the solution was determined, and the SCN^−^ content was calculated.

## 3 Results and discussion

### 3.1 Experimental study on the adsorption of Au(CN)_2_^-^ by D301 resin

To investigate the effect of SCN^−^ on the adsorption efficiency of Au(CN)_2_^-^ by D301 resin, this section systematically examined the influence of factors such as adsorption experimental temperature, initial solution pH, and resin dosage on the adsorption of Au(CN)_2_^-^ by D301 resin, both in the presence and absence of SCN^−^.

It should be noted that the adsorption experiments in Sections 3.1–3.3 were conducted using synthetic solutions containing Au(CN)_2_^-^, CN^−^, and SCN^−^ as the primary components, rather than actual industrial liquors which typically contain various competing metal–cyanide complexes and anions. Therefore, the findings from these sections should be interpreted with this limitation in mind.

#### 3.1.1 Influence of different factors on the adsorption efficiency of Au(CN)_2_^-^ by D301 resin in the absence of SCN^−^

**FIGURE 6:**
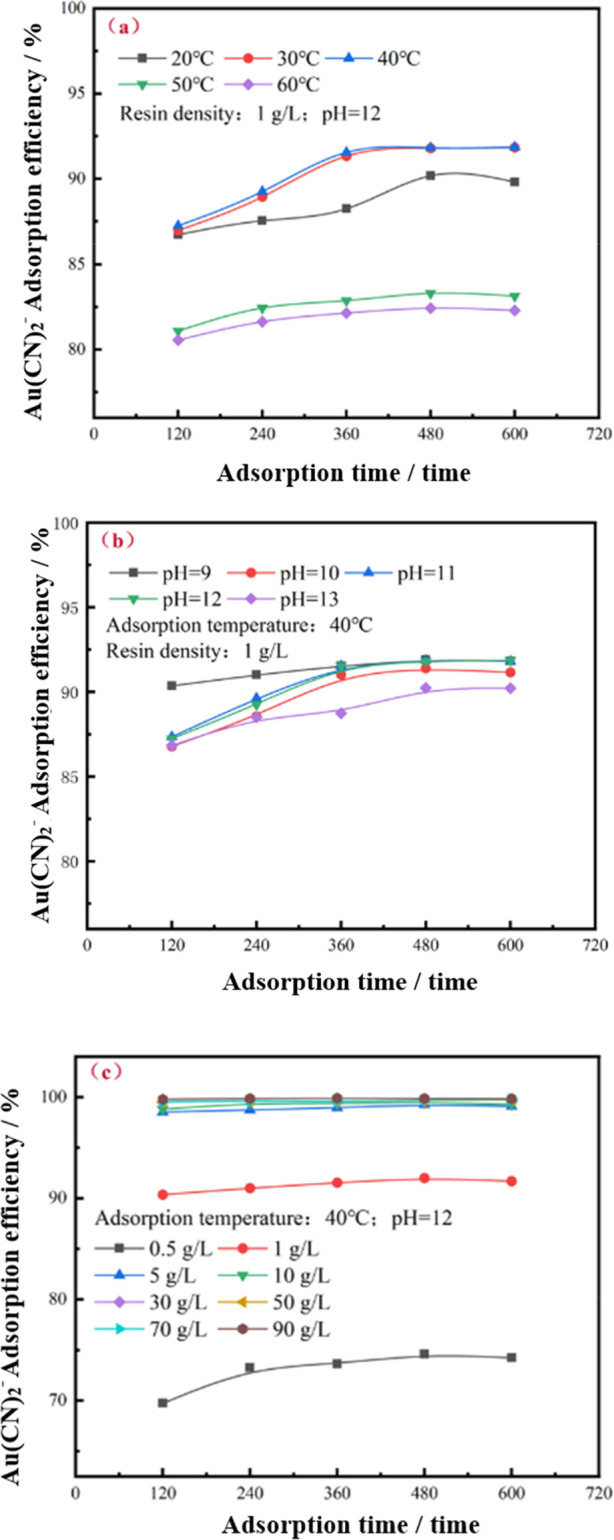
Effects of conditions on Au(CN)_2_^-^ adsorption onto D301 resin without SCN^−^. **(a)** Different adsorption temperature. **(b)** Different initial pH. **(c)** Different resin density.

The effects of adsorption temperature, initial solution pH, and resin dosage on Au(CN)_2_^-^ adsorption were investigated in SCN^−^-free solutions. All experiments were conducted with initial Au(CN)_2_^-^ and CN^−^ concentrations of 35 mg/L and 2000 mg/L, respectively. The adsorption efficiency was calculated using the equation in Section 2.2, and the results are presented in Figure 6.

As shown in Figure 6a, the adsorption curves at different temperatures exhibited similar trends: adsorption efficiency increased with time and then plateaued, indicating saturation. The optimum temperature was 40 °C, at which the adsorption efficiency reached 87.20% at 120 min and 91.82% at 480 min. For a given adsorption time, efficiency first increased and then decreased with rising temperature. This is attributed to the exothermic nature of most adsorption reactions: moderate heating accelerates molecular exchange and promotes adsorption, whereas excessive temperature impedes the forward reaction, thereby reducing efficiency.

As shown in Figure 6b, adsorption curves under different initial pH conditions followed a similar pattern over time. The highest efficiency was achieved at pH 9, reaching 90.36% at 120 min and 91.89% at 480 min. Overall, adsorption efficiency showed a decreasing trend with increasing initial pH. Two factors account for this: (i) D301 is a weakly basic anion exchange resin, and its functional groups become partially deactivated when the pH exceeds the pKa, reducing Au(CN)_2_^-^ uptake; and (ii) high OH^−^ concentrations may displace adsorbed Au(CN)_2_^-^ from the resin. Measurements of post-adsorption pH showed that the final pH remained around 9.00–9.50 except for the initial pH 9 condition, where it dropped to approximately 8.00, indicating that higher initial pH leads to more OH^−^ adsorption and stronger competitive effects.

As shown in Figure 6c, adsorption efficiency at low resin density (dosages of 0.5–1.0 g/L) varied significantly over time, whereas at high dosages (≥5.0 g/L) it increased more rapidly and reached equilibrium sooner. At 5.0 g/L, the efficiency reached 98.53% at 120 min and 99.25% at 480 min. At a given adsorption time, efficiency increased with resin dosage and then levelled off. This is explained by the saturation capacity of D301 resin (approximately 32.20 mg Au(CN)_2_^-^ per Gram). With an initial Au(CN)_2_^-^ concentration of only 35 mg/L, dosages below 1.0 g/L were limiting; above this threshold, the Au(CN)_2_^-^ concentration became the limiting factor, causing the adsorption efficiency to stabilise.

#### 3.1.2 Influence of various factors on the adsorption efficiency of Au(CN)_2_^-^ by D301 resin in the presence of SCN^−^

**FIGURE 7:**
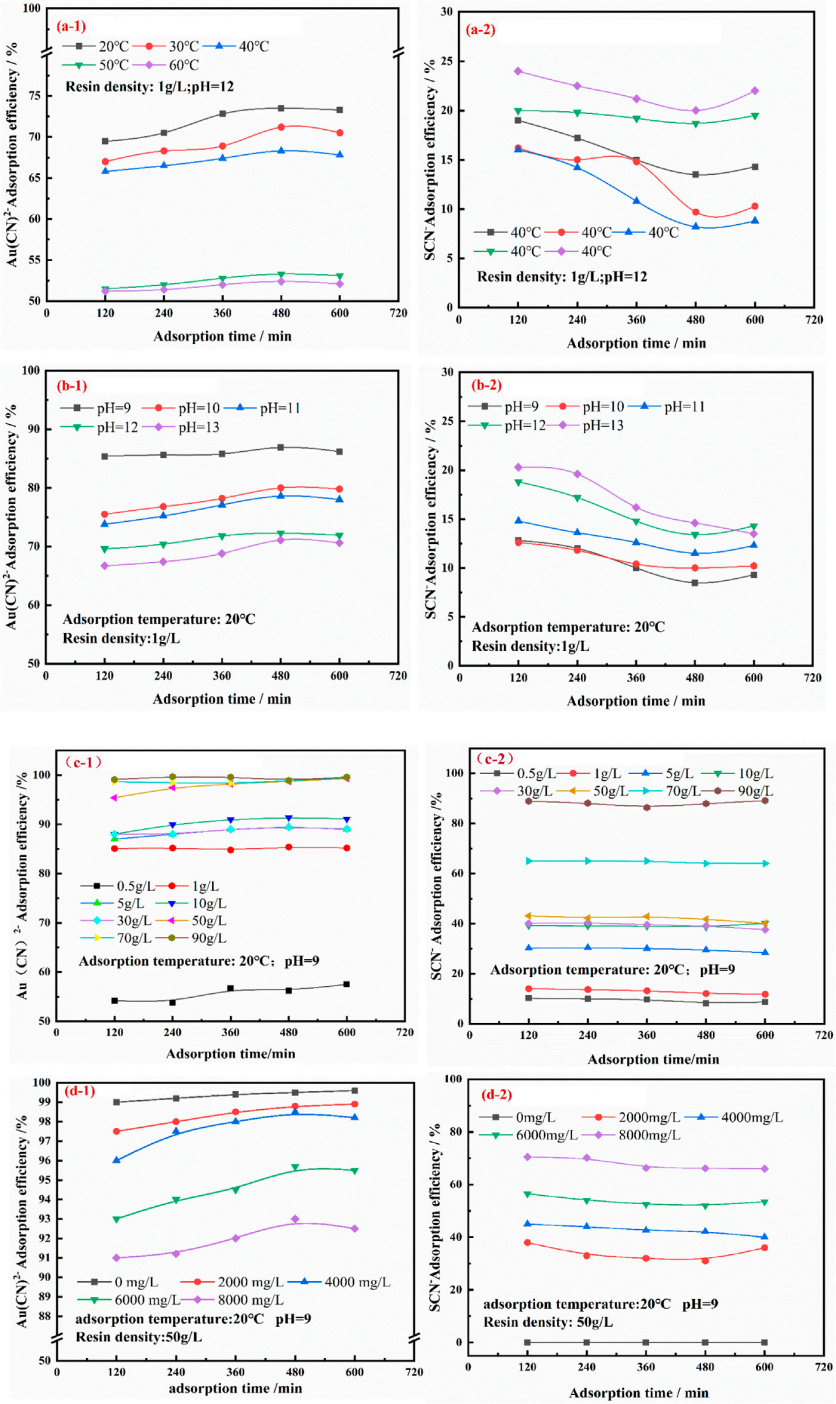
Influence of experimental conditions on the adsorption of Au(CN)_2_^-^ by D301 resin. **(a-1)** Different adsorption temperature. **(a-2)** Different adsorption temperature. **(b-1)** Different initial pH. **(b-2)** Different initial pH. **(c-1)** Different resin density 100. **(c-2)** Different resin density. **(d-1)** SCN concentration. **(d-2)** SCN concentration.

The effects of adsorption temperature, initial solution pH, resin dosage, and SCN^−^ concentration on Au(CN)_2_^-^ adsorption were examined in the presence of SCN^−^. Except for the SCN^−^ concentration trials, a fixed SCN^−^ concentration of 4,000 mg/L was used. All experiments were performed with initial Au(CN)_2_^-^ and CN^−^ concentrations of 35 mg/L and 2000 mg/L, respectively. The results are presented in Figure 7.

As shown in Figure 7a, at different temperatures, Au(CN)_2_^-^ adsorption efficiency increased with time before reaching equilibrium, whereas SCN^−^ adsorption efficiency decreased initially and then rose slowly. This opposite trend indicates competitive adsorption between the two species. The highest Au(CN)_2_^-^ adsorption was observed at 20 °C, reaching 69.46% at 120 min and 72.03% at 480 min. Increasing temperature markedly reduced Au(CN)_2_^-^ adsorption while SCN^−^ adsorption first decreased and then increased. Overall, adsorption of both species was exothermic. Above 40 °C, however, this trend reversed, possibly due to enhanced SCN^−^ mobility that may displace pre-adsorbed Au(CN)_2_^-^. Direct evidence for this desorption effect is not yet available and requires verification through independent experiments. Consequently, Au(CN)_2_^-^ adsorption decreased while SCN^−^ uptake increased, indicating that SCN^−^ significantly alters the adsorption behavior.

As shown in Figure 7b, Au(CN)_2_^-^ adsorption efficiency increased with time and then plateaued under different pH conditions, while SCN^−^ adsorption showed the opposite trend. The optimum was observed at pH 9, with Au(CN)_2_^-^ adsorption reaching 85.38% at 120 min and 86.88% at 480 min. For a given adsorption time, increasing initial pH progressively reduced Au(CN)_2_^-^ adsorption but enhanced SCN^−^ uptake. The strongly alkaline environment not only reduces the resin’s intrinsic adsorption capacity but also promotes competitive adsorption from OH^−^ and SCN^−^. Measurements showed that the post-adsorption pH dropped by approximately 1.50 units relative to the initial pH, suggesting that SCN^−^ may act as a buffer for the acid–base equilibrium in the system.

As shown in Figure 7c, Au(CN)_2_^-^ and SCN^−^ adsorption exhibited distinct competitive behaviour with varying resin density (here referring to resin dosage). As time progressed, SCN^−^ adsorption gradually decreased while Au(CN)_2_^-^ adsorption continued to rise until equilibrium. Saturation was achieved at a resin dosage of 50 g/L, with Au(CN)_2_^-^ adsorption reaching 95.40% at 120 min and 98.75% at 480 min. At a given adsorption time, increasing resin dosage improved both species’ adsorption, but above 50 g/L, further dosage increases mainly enhanced SCN^−^ uptake while Au(CN)_2_^-^ adsorption increased only marginally. This indicates that at low resin dosages, Au(CN)_2_^-^ adsorption is significantly hindered by competition; at high dosages, Au(CN)_2_^-^ is preferentially adsorbed, with residual sites occupied by SCN^−^.

As shown in Figure 7d, at different SCN^−^ concentrations, both Au(CN)_2_^-^ and SCN^−^ adsorption approached equilibrium over time. At a given adsorption time, increasing SCN^−^ concentration progressively reduced Au(CN)_2_^-^ adsorption but enhanced SCN^−^ uptake. This confirms that SCN^−^ exerts a pronounced competitive effect on Au(CN)_2_^-^ adsorption, which intensifies with increasing SCN^−^ concentration.

#### 3.1.3 Comparative adsorption of Au(CN)_2_^-^ with and without SCN^−^

**FIGURE 8:**
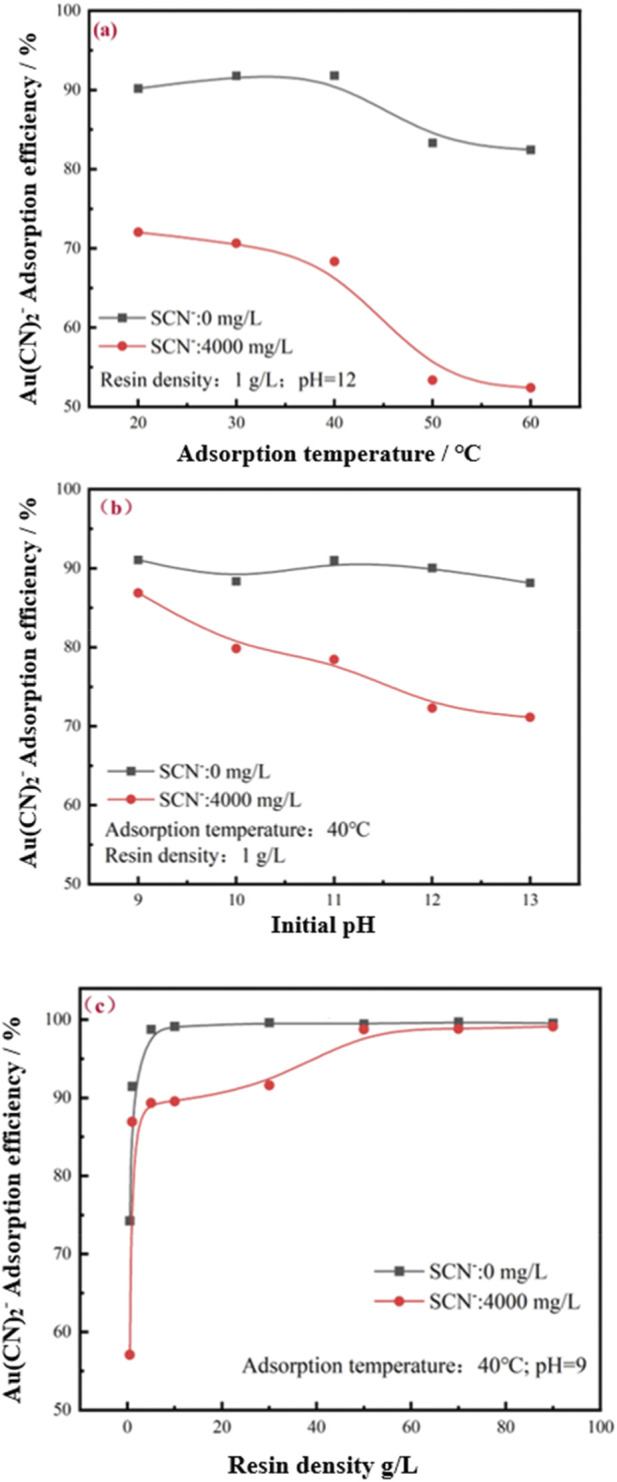
Comparative experimental results of different adsorption factors on resin adsorption of Au(CN)_2_^-^ in the presence and absence of SCN^−^. **(a)** Different adsorption temperature. **(b)** Different initial pH. **(c)** Different resin density.

As demonstrated in Sections 3.1.1, 3.1.2, the presence of SCN^−^ significantly affects Au(CN)_2_^-^ adsorption onto D301 resin. To further elucidate this effect, comparative experiments with and without SCN^−^ were conducted under varying adsorption temperature, initial solution pH, and resin dosage. All tests were performed at an adsorption time of 480 min to ensure equilibrium. The results are presented in Figure 8.

As shown in Figure 8a, regardless of SCN^−^ presence, the adsorption efficiency of Au(CN)_2_^-^ first decreases and then stabilises with increasing temperature. In the SCN^−^-containing system, the Au(CN)_2_^-^ adsorption efficiency peaks at 72.03% at 20 °C, where high SCN^−^ concentrations compete with Au(CN)_2_^-^ for adsorption sites. Resin saturation occurs at lower temperatures; heating not only diminishes Au(CN)_2_^-^ adsorption capacity but may also promote displacement of adsorbed Au(CN)_2_^-^ by SCN^−^, potentially causing partial desorption. This possible displacement effect, however, needs to be confirmed by dedicated desorption experiments with pre-loaded resin. Concurrently, Figure 8a demonstrates markedly higher Au(CN)_2_^-^ adsorption efficiency in the absence of SCN^−^, indicating that SCN^−^ presence impedes Au(CN)_2_^-^ adsorption. In summary, the presence of SCN^−^ significantly alters the adsorption characteristics of D301 resin. Without SCN^−^, the adsorption efficiency increases during the initial heating phase. However, in the presence of SCN^−^, heating intensifies competitive adsorption and desorption processes, leading to a marked reduction in Au(CN)_2_^-^ adsorption efficiency.

As shown in Figure 8b, adsorption efficiency in both systems decreased with increasing initial pH, with the optimum at pH 9 (91.05% without SCN^−^ vs. 86.88% with SCN^−^). This decline is attributed to partial deactivation of the weakly basic resin at high pH and competitive adsorption from OH^−^. The reduction was more pronounced in the presence of SCN^−^, suggesting that SCN^−^ and OH^−^ may exert synergistic competitive effects, and potentially enhance the displacement of adsorbed Au(CN)_2_^-^. Consequently, the initial solution pH exerts a stronger influence on Au(CN)_2_^-^ adsorption when SCN^−^ is present, with competitive and possible desorption effects being contributing factors to the reduced adsorption performance.

As shown in Figure 8c, adsorption efficiency in both systems increased with resin density (referring to resin dosage) and then plateaued. At low dosages, SCN^−^ markedly suppressed Au(CN)_2_^-^ adsorption, but this difference narrowed at higher dosages. In the SCN^−^-free system, saturation was reached at 5 g/L (99.25%), whereas in the SCN^−^-containing system, 50 g/L was required to achieve a comparable efficiency (98.75%). This demonstrates that while SCN^−^ significantly impairs Au(CN)_2_^-^ adsorption, its adverse effect can be effectively mitigated by increasing resin dosage.

### 3.2 Analysis of SEM-EDS test results

**FIGURE 9:**
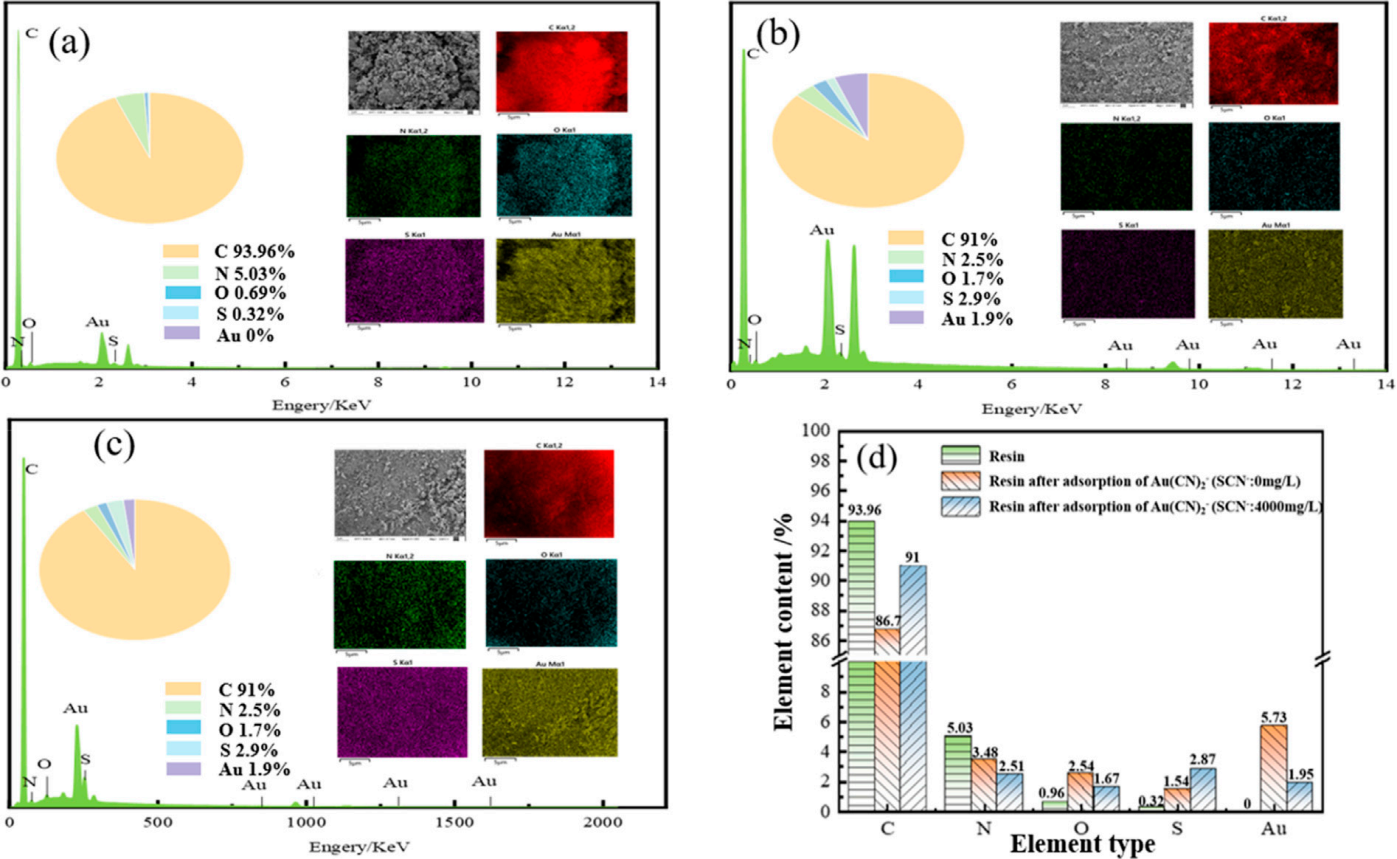
SEM-EDS results of **(a)** fresh resin, **(b)** after adsorption without SCN^−^, **(c)** after adsorption with SCN^−^, and **(d)** a comparative overview of the elemental distributions.

For SEM-EDS analysis, fresh resin and resin samples after adsorption in SCN^−^-free and SCN^−^-containing solutions were ground, dried, and examined. It should be noted that SEM-EDS is a semi-quantitative and surface-sensitive technique, and the elemental contents obtained reflect the resin surface rather than the bulk loading. The corresponding spectra and elemental distributions are presented in Figure 9: (a) fresh resin, (b) after adsorption without SCN^−^, (c) after adsorption with SCN^−^, and (d) a comparative overview of the elemental distributions.

As shown in Figure 9, the EDS spectra and elemental distributions of the resin differed markedly after adsorption in SCN^−^-free versus SCN^−^-containing solutions. Prior to adsorption, no Au was detected on the fresh resin. After adsorption, the near-surface Au content increased to 5.73% in the SCN^−^-free system, but only to 1.95% in the presence of SCN^−^ (as determined by semi-quantitative EDS analysis). This substantial decrease indicates that SCN^−^ significantly inhibits Au(CN)_2_^-^ adsorption onto the resin. However, because EDS is a surface-sensitive and semi-quantitative technique, these values reflect the elemental composition of the resin surface rather than the total bulk gold loading. In addition, the resin adsorbed in the SCN^−^-containing solution showed higher C and S contents compared with both the fresh resin and the resin adsorbed without SCN^−^, confirming that SCN^−^ was also adsorbed onto the resin via ion exchange.

### 3.3 Analysis of XRD test results

**FIGURE 10:**
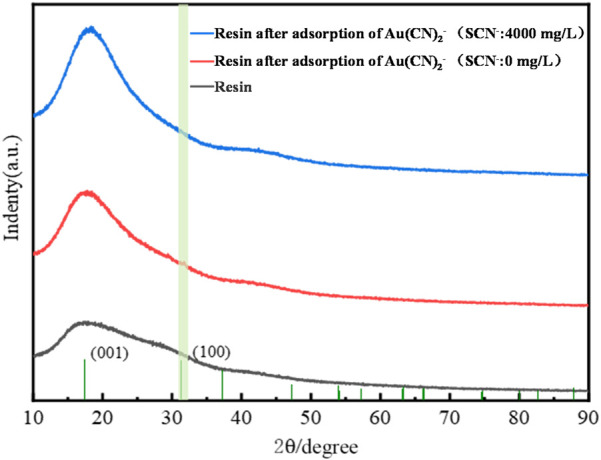
XRD patterns of the resin before and after adsorption.

To analyse the phase changes in the resin before and after adsorption of Au(CN)_2_^-^, XRD analysis was conducted on resin powder samples prior to adsorption and following adsorption under different solution conditions. The results are presented in Figure 10.

As shown in Figure 10, the XRD patterns of the resin before and after adsorption under different solution conditions exhibit only broad halo peaks characteristic of amorphous polymeric materials, with no distinct diffraction peaks attributable to Au(CN)_2_^-^ or other crystalline phases. This is consistent with the amorphous nature of the resin matrix and the relatively low gold loading on the resin. No meaningful conclusions regarding the crystallinity or structural state of the adsorbed species can be drawn from these patterns. Therefore, the adsorption mechanism and changes in resin structure were further investigated using FTIR and XPS, as discussed in the following sections.

### 3.4 Analysis of FTIR test results

**FIGURE 11:**
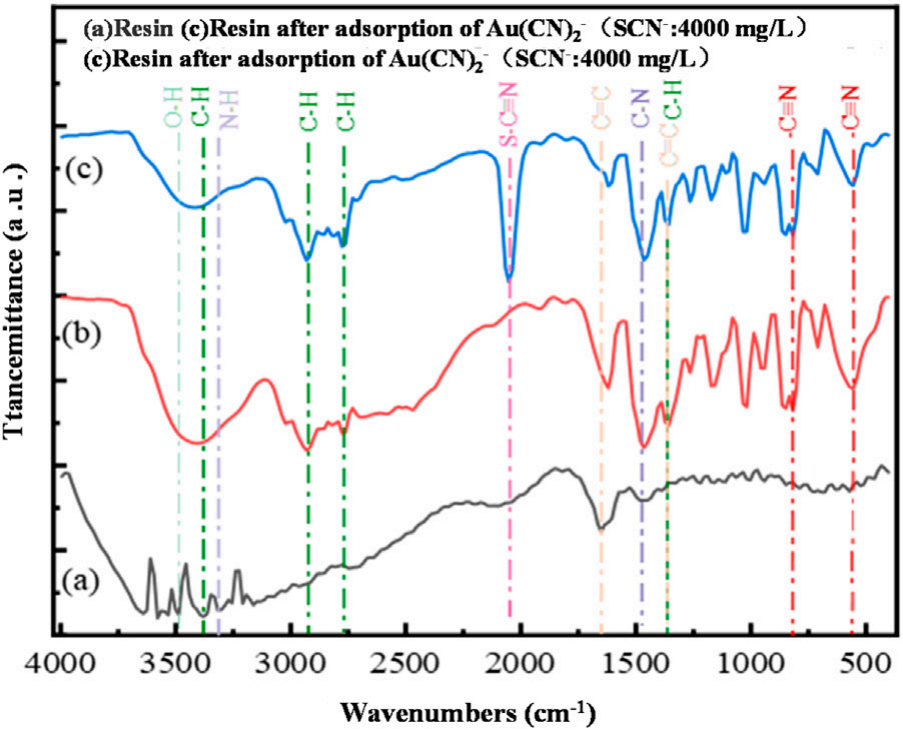
FTIR test results of resin under different adsorption systems.

To investigate the functional group transformations accompanying Au(CN)_2_^-^ adsorption and to evaluate the impact of SCN^−^ on the adsorption process, FTIR spectroscopy was carried out on fresh D301 resin and on resin samples recovered after adsorption in both the absence and presence of SCN^−^. The corresponding spectra are displayed in Figure 11.

As shown in Figure 11, the fresh resin displays absorption bands at 3,486.66, 3,363.24, and 1,650.77 cm^−1^, assigned to O–H stretching of adsorbed water, C–H stretching of the benzene ring, and C=C stretching of the benzene ring, respectively. After adsorption, both systems exhibit a broad band at 3,300–3,600 cm^−1^, which is attributed to O–H stretching of residual water rather than to N–H vibrations, as D301 is a tertiary amine resin (Musto et al., 2003; OpenStax, 2023). In the SCN^−^-free system, four new peaks appear at 2,931.27, 2,761.54, 1,465.63, and 1,357.64 cm^−1^, assigned to C–H vibrations of–CH_2_– and N–CH_3_, C–N stretching of–N^+^(CH_3_)_2_, C–H deformations, and benzene ring C=C stretching. These peaks shift to 2,946.69, 2,776.99, 1,450.20, and 1,357.64 cm^−1^ in the presence of SCN^−^, indicating the involvement of these groups in Au(CN)_2_^-^ adsorption.

Notably, two new peaks at 709.68 and 555.39 cm^-1^ are observed in the SCN^−^-free system. These are not C≡N stretching vibrations (which typically appear at 2,140–2,160 cm^−1^) but rather Au–C or Au–N vibrations, confirming Au(CN)_2_^-^ adsorption. The more pronounced intensity changes in the SCN^−^-free system indicate stronger adsorption. By contrast, the resin adsorbed in the SCN^−^-containing solution shows a prominent peak at 2067.32 cm^−1^, assignable to the S–C≡N stretching of SCN^−^, confirming substantial SCN^−^ uptake. This demonstrates that SCN^−^ competes with Au(CN)_2_^-^ for adsorption sites, thereby significantly reducing the resin’s efficiency for Au(CN)_2_^-^ adsorption.

### 3.5 Analysis of XPS test results

**FIGURE 12:**
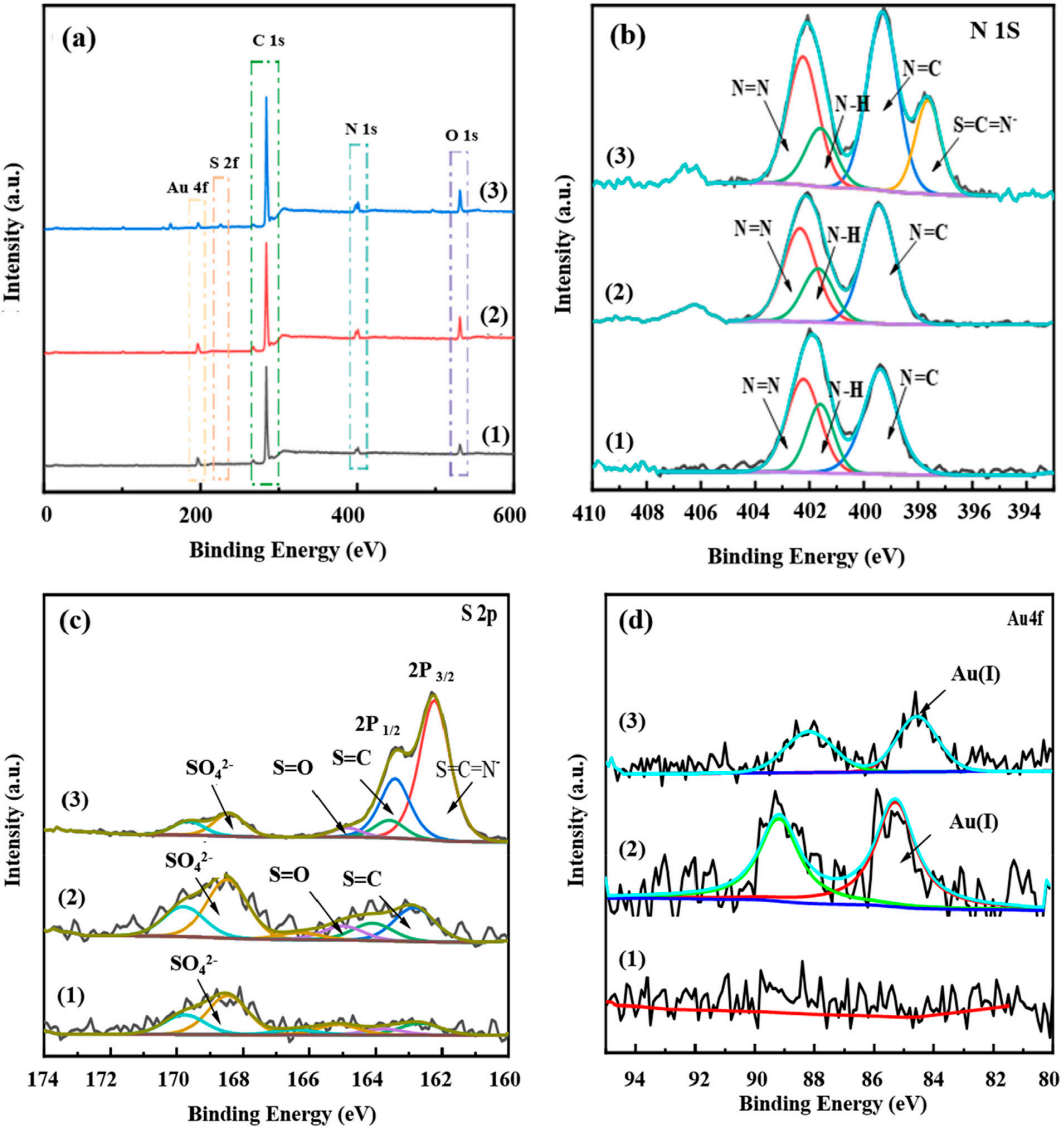
Peak-deconvoluted XPS spectra of D301 resin under different adsorption conditions: **(a)** Survey spectrum; **(b)** N 1s spectrum; **(c)** S 2p spectrum; **(d)** Au 4f spectrum. (1) Fresh D301; (2) Au(CN)_2_^-^ adsorbed (SCN^-^:0 mg/L) (3) Au(CN)_2_^-^ adsorbed (SCN^-^:4000 mg/L).

To further investigate changes in the surface elemental composition and valence states of the target elements on the resin surface before and after adsorption of Au(CN)_2_^-^ under different adsorption solution conditions, this section employs XPS characterisation of the resin prior to adsorption and after adsorption under various solution environments. The results of peak fitting analysis for the overall XPS spectrum and individual elemental spectra are presented in Figures 12a–d.

As shown in Figure 12a, both adsorption systems exhibited characteristic Au photoelectron peaks after adsorption, along with enhanced C and N signals, indicating that Au was adsorbed onto D301 resin in the form of Au(CN)_2_^-^. In the presence of SCN^−^, an additional S peak appeared, and the C and N signals became more pronounced compared to the SCN^−^-free system, confirming the co-adsorption of SCN^−^ onto the resin—a finding consistent with the preceding experimental results.

As shown in Figure 12b, the intensity of the N-H adsorption peak in the post-adsorption resin weakened under both adsorption solution conditions compared to the pre-adsorption resin, indicating that the adsorption of Au(CN)_2_^-^ onto the resin resulted from -NR_2_ interactions. However, in the presence of high SCN^−^ concentrations, a prominent absorption peak attributable to S=C=N^−^ appeared, confirming the adsorption of SCN^−^ onto the resin. Peak fitting of the N 1s spectrum (Figure 12b) yielded components assignable to N–H and N=C species. Additionally, the appearance of the N=N peak may arise from side reactions or impurities during sample preparation.

As shown in Figure 12c, in the presence of high SCN^−^ concentrations, a minor portion of SCN^−^ undergoes hydrolysis, giving rise to peaks attributable to SO_4_^2-^ and S-O species. Nevertheless, SCN^−^ is predominantly adsorbed onto the resin surface in the form of S=C=N^−^. As shown in Figure 12d, the Au 4f spectrum of the resin after Au(CN)_2_^-^ adsorption was deconvoluted into two characteristic peaks, both assigned to Au(I) in Au(CN)_2_^-^, indicating that the valence state of gold remained unchanged during adsorption. However, in the SCN^−^-containing system, the Au 4f peaks shifted and exhibited lower intensity compared with those in the SCN^−^-free system, clearly demonstrating that the presence of SCN^−^ adversely affects Au(CN)_2_^-^ adsorption onto the resin.

**FIGURE 13:**
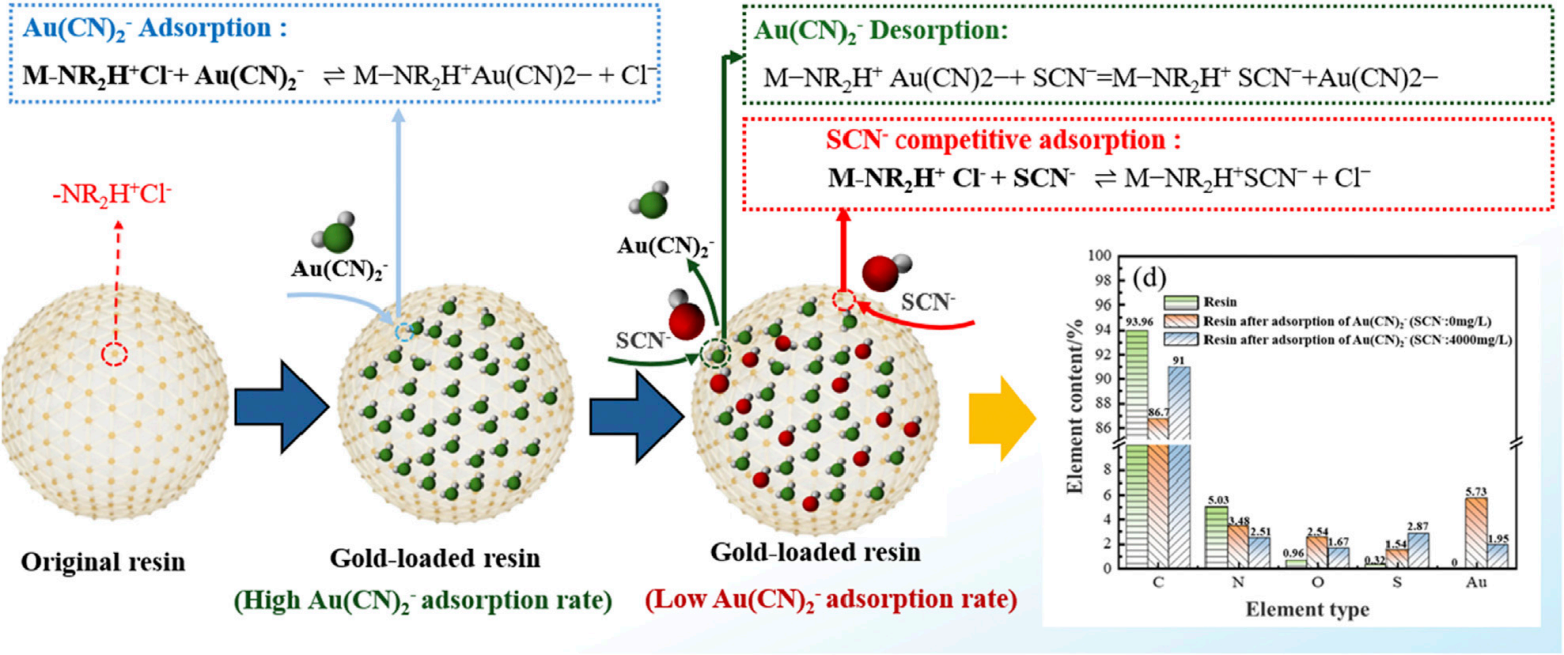
Schematic diagram of the adsorption model of SCN^−^ and Au(CN)_2_^-^ on D301 resin.

In summary, based on the aforementioned mechanistic testing and analysis, during the gold cyanidation leaching process, SCN^−^ ions hinder the adsorption of Au(CN)_2_^-^ onto the resin through two concurrent actions: competitive adsorption with Au(CN)_2_^-^ and desorption of already-loaded Au(CN)_2_^-^. Consequently, the resin’s Au(CN)_2_^-^ adsorption efficiency is significantly reduced. To improve recovery efficiency, it is therefore essential to remove SCN^−^ prior to or during the adsorption process. The adsorption model of SCN^−^ on the resin is illustrated in Figure 13.

### 3.6 Oxidative removal of SCN^−^ and its effect on Au(CN)_2_^-^ adsorption by resin

**FIGURE 14:**
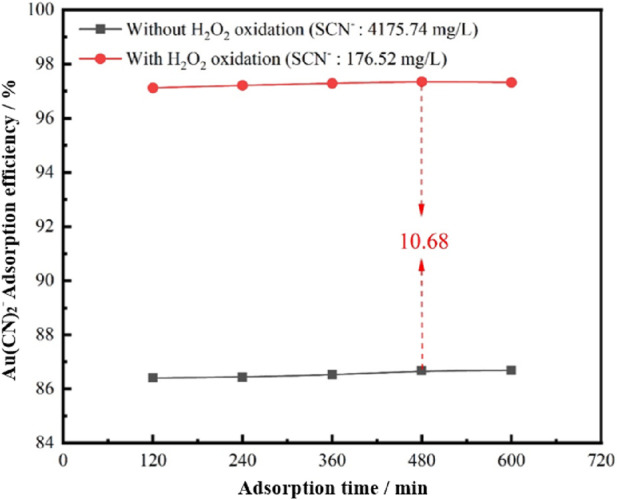
Comparison of Au(CN)_2_^-^ adsorption onto D301 resin before and after oxidative removal of SCN^−^ from the circulating solution of the Chifeng Gold Mine, Inner Mongolia, China.

The preceding study has demonstrated that the presence of SCN^−^ significantly reduces the Au(CN)_2_^-^ adsorption rate onto the resin during cyanide leaching. To mitigate this issue, this section investigates the circulating water from a gold cyanidation plant in Chifeng, Inner Mongolia, China, which contains 4,175.74 mg/L SCN^−^. H_2_O_2_ oxidation experiments were conducted on this water to determine both the SCN^−^ removal efficiency and the subsequent Au(CN)_2_^-^ adsorption performance of D301 resin. Under the optimized conditions (200 mL of water, oxidation at 30 °C for 60 min, and an H_2_O_2_ dosage of 25% by volume), the SCN^−^ removal efficiency reached 96.14%, leaving a residual SCN^−^ concentration of only 176.52 mg/L. The adsorption capacity of D301 resin for Au(CN)_2_^-^ was then evaluated using the oxidized water, with each adsorption test performed in triplicate and the averaged results reported. A comparative analysis of resin adsorption before and after SCN^−^ oxidation removal is presented in Figure 14.

As shown in Figure 14, prior to oxidative removal of SCN^−^ (at an initial concentration of 4,175.74 mg/L), the resin exhibited an Au(CN)_2_^-^ adsorption efficiency of only 86.67% after 480 min. Following oxidation, with the SCN^−^ concentration reduced to 176.52 mg/L, the adsorption efficiency increased markedly to 97.35% under the same conditions. This demonstrates that oxidative removal of SCN^−^ from cyanide gold leaching solutions substantially enhances the resin’s Au(CN)_2_^-^ adsorption capacity, effectively overcoming the problem of low gold loading caused by high SCN^−^ levels in the circulating water of resin-in-pulp operations, and thereby significantly improving resin utilization efficiency. While H_2_O_2_ oxidation effectively enhanced Au(CN)_2_^-^ adsorption, the long-term stability and reusability of the resin under repeated oxidative conditions require further investigation in future work.

## 4 Conclusion

This study examined the effects of SCN^−^ on Au(CN)_2_^-^ adsorption onto D301 resin using single-factor and comparative experiments, with the interference eliminated via H_2_O_2_ oxidation. The main findings are as follows:

1. In SCN^−^-free solutions, Au(CN)_2_^-^ adsorption efficiency increased with time to a maximum of 91.82% at 40 °C (480 min), varied with pH (optimum 91.89% at pH 9), and reached saturation at 5 g/L resin dosage (99.25%).
2. In SCN^−^-containing solutions, adsorption showed similar trends but lower efficiencies: 72.03% at 20 °C, 86.88% at pH 9, and 98.75% at 50 g/L resin dosage. Increasing SCN^−^ from 0 to 8,000 mg/L reduced Au(CN)_2_^-^ adsorption from 99.45% to 92.83%.
3. SCN^−^ markedly reduced Au(CN)_2_^-^ adsorption capacity while preserving the overall response trends to temperature, pH, and resin dosage. This negative effect can be mitigated by lowering SCN^−^ concentration or increasing resin dosage.
4. Characterization confirmed competitive adsorption between SCN^−^ and Au(CN)_2_^-^: SEM-EDS surface analysis showed Au content decreasing from 5.73% to 1.95% with SCN^−^ present; FTIR exhibited weakened Au(CN)_2_^-^ peaks and emerging S–C≡N bands; XPS revealed enhanced C/N/S signals and a reduced Au peak. Indirect evidence also suggests a possible displacement of pre-adsorbed Au(CN)_2_^-^ by SCN^−^, which requires direct experimental verification.
5. H_2_O_2_ oxidation effectively removed SCN^−^ from industrial circulating water (initial 4,175.74 mg/L), raising Au(CN)_2_^-^ adsorption efficiency from 86.67% to 97.35%, thus substantially improving resin utilization.

## Data Availability

The original contributions presented in the study are included in the article/supplementary material, further inquiries can be directed to the corresponding authors.
